# Disparities in adult women's access to contraception during COVID-19: a multi-country cross-sectional survey

**DOI:** 10.3389/fgwh.2024.1235475

**Published:** 2024-12-24

**Authors:** Sara Cavagnis, Rebecca Ryan, Aamirah Mussa, James R. Hargreaves, Joseph D. Tucker, Chelsea Morroni

**Affiliations:** ^1^ School of Hygiene and Preventive Medicine (Department of Biomedical and Neuromotor Sciences), University of Bologna, Bologna, Italy; ^2^ Botswana Sexual and Reproductive Health Initiative, Botswana Harvard AIDS Institute Partnership, Gaborone, Botswana; ^3^ Department of Epidemiology and Evaluation, London School of Hygiene & Tropical Medicine, London, United Kingdom; ^4^ Institute of Global Health and Infectious Diseases, University of North Carolina at Chapel Hill, Chapel Hill, NC, United States; ^5^ Clinical Research Department, Faculty of Infectious and Tropical Diseases, London School of Hygiene and Tropical Medicine, London, United Kingdom; ^6^ MRC Centre for Reproductive Health, University of Edinburgh, Edinburgh, United Kingdom

**Keywords:** contraception, family planning, COVID-19, disparities, access, income loss

## Abstract

During the COVID-19 pandemic, family planning services over the world have been disrupted. There are still uncertainties about the impact on access to contraception, particularly among marginalised populations. This study aimed to assess the effect of COVID-19 on women's access to contraception, focusing on those experiencing loss of income and self-isolation. The International Sexual Health and Reproductive Health (I-SHARE) survey collected data from 5,216 women in 30 countries. Multivariable logistic regression was conducted to assess the association between loss of income during the pandemic, self-isolation and reduced access to contraception. Women experiencing loss of income and those who had self-isolated had reduced access to contraception (respectively aOR 2.3 and 1.7, for both *p* < 0.001). Most women reported inaccessibility of health centres, fear of COVID-19, and stockouts as reasons for reduced access. This study highlights how socio-demographic differences may have impacted access to contraception during the pandemic. People experiencing income loss and self-isolation might have faced increased barriers to family planning during the pandemic. Contraception should be prioritised in times of crisis: when planning services, financial support, telehealth and other measures should be implemented in order to increase access and reduce inequalities.

## 1 Introduction

Access to contraception is central to reducing poverty, empowering women and achieving sustainable development. The COVID-19 pandemic limited people's timely access to contraception (1, 2). Both COVID-19 itself and the public health measures implemented have weakened economies and destabilised health systems; health disparities have increased (3). According to the World Economic Forum, one in two people lost income during COVID-19 (4). Family planning services have been disrupted because many health facilities closed and healthcare professionals focused on COVID-19. In addition, people were unable to access health services because of travel restrictions, unaffordability and fear of COVID-19 (5). Telehealth and related adaptations have been variably implemented.

More research is needed on the pandemic's impact on already underserved populations, and on the mechanisms that led to reduced access (1). This analysis aimed to assess women's access to contraception during the pandemic at a multi-country level, focusing on those experiencing loss of income and self-isolation.

## 2 Methods

We used data from the International Sexual Health And REproductive health (I-SHARE) survey, a cross-sectional online survey conducted between 2020 and 2021 in 30 countries. There were 23,067 respondents to the survey. Sampling methods varied between countries and included convenience sampling, population-representative samples, and online panels (23, 2 and 6 countries respectively; one country used two methods). Details on the survey methods have been published (6) and the survey instrument, the countries included, their sample size and sampling strategy are attached in the Supplementary Materials.

For this paper, we restricted the analyses to participants who were assigned female sex at birth, younger than 50 years old. Participants were asked about their pregnancy intentions: we included only those who selected the option “Not currently pregnant and don't wish to be in the near future”. We excluded participants who reported having reached menopause, those with fertility issues, and those who were not sexually active. We also excluded participants who reported that they hadn't needed to seek or obtain contraceptives during the pandemic, and those with missing data on the outcome of access to contraception. The final population for this analysis included 5,216 participants (flowchart of participant inclusion in Supplementary Materials). Included participants were mainly from Europe (55.1%) and Latin America and the Caribbean (34.6%).

Reduced access to contraception was defined as a positive answer to the question “Have the COVID-19 social distancing measures stopped or hindered you from seeking or obtaining contraception?”. The two main exposure variables investigated were personal loss of income and self-isolation due to COVID-19.

We used multivariable logistic regression to explore the association between the exposures of interest and the outcome. Country of residence was included *a priori* in the regression models as a fixed-effect; to adjust for confounding for socio-demographic variables, we used a forward causal modelling strategy with a change-in-estimate approach. We introduced one by one in the model the variables that were associated with both exposure and outcome in the bivariable analysis, and then kept in the model the confounder that changed the OR more. We repeated this process until the OR for the exposure didn't change any more. Missing data was treated by pairwise deletion (available-case analysis).

## 3 Results

The analysed population was predominantly young, heterosexual, cisgender and highly educated; more than half were in a relationship (Table 1). Overall, around a third of the participants reported losing income during the pandemic. One in four had isolated due to COVID-19.

**Table 1:** Socio-demographic characteristics of the participants included in this analysis (*N* = 5,216). The percentages only apply to non-missing data out of the total study population. I-SHARE online survey 2020/21.

		*N*	%
Age group in years	18–24	1,705	32.7
	25–29	1,477	28.3
	30–39	1,431	27.4
	40–49	603	11.6
Sexual orientation	Heterosexual	4,160	82.6
	Non-heterosexual	873	17.4
	Missing	183	
Gender identity	Cisgender	4,763	91.2
	Non-cisgender/other^a^	458	8.8
	Missing	22	
Education	Primary education or less	208	4.0
	Some/completed secondary education	1,010	19.4
	Some/completed college or university	3,849	73.8
	Other^a^	148	2.8
	Missing	1	
Relationship status	Married	1,091	20.9
	In a relationship	2,780	53.4
	Single	1,191	22.9
	Other^b^	145	2.8
	Missing	9	
Household income position before COVID-19	Lower than average	1,601	34.7
	Average	1,721	37.3
	Higher than average	1,289	27.9
	Missing	605	
Employment status before COVID-19	Employed or self-employed	3,063	58.7
	Informal work or unemployed	362	6.9
	Student	1,110	21.3
	Other^b^	681	13.1
Change in personal income during COVID-19	No loss of income	2,594	51.6
	Yes, total loss of income	364	7.2
	Yes, partial loss of income	1,264	25.1
	No personal income before COVID-19	805	16.0
	Missing	189	
Ever been in isolation due to COVID-19	No	3,826	73.4
	Yes	1,387	26.6
	Missing	3	

^a^ People selecting “Other” could not specify further.

^b^ Includes people selecting multiple options and people selecting “Other” (which could not be specified further).

Overall, 9.1% of the respondents (476/5,216) reported being stopped from seeking or accessing contraception due to COVID-19. Most reported inaccessibility of health centres, fear of COVID-19 and stockouts as reasons for reduced access.

After adjusting for country, age and household income position before COVID-19, those who had experienced a total loss of income had twice the odds of reporting reduced access to contraception compared to those who had no loss of income (aOR 2.3, 95% CI: 1.7–3.3, *p* < 0.001). Those experiencing a partial loss of income also had higher odds of reduced access to contraception (aOR 1.3, 95% CI: 1.0–1.7, *p* = 0.04) (Table 2).

**Table 2:** Association between the two main exposures and reduced access to contraception. I-SHARE online survey 2020/21.

	N cases (row%)	OR (95% CI)*	Fully adjusted OR (95% CI)**	*p* value***
Loss of income (*N* = 4,457)				
No loss of income	160 (6.4)	1	1	
Yes, total loss	66 (19.0)	2.8 (2.0–3.9)	2.3 (1.7–3.3)	<0.001
Yes, partial loss	112 (9.8)	1.5 (1.1–1.9)	1.3 (1.0–1.7)	0.04
No personal income before COVID-19	68 (10.3)	1.4 (1.0–1.9)	1.0 (0.7–1.4)	0.86
Isolation due to COVID-19 (*N* = 4,609)				
No	280 (8.3)	1	1	
Yes	152 (12.5)	1.6 (1.3–2.0)	1.7 (1.3–2.1)	<0.001

* Adjusted for country. The ORs were calculated on the same population as the aORs.

** Adjusted for country, age class and household income position before COVID-19 for loss of income; adjusted for country, employment status before COVID-19 and household income before COVID-19 for isolation.

*** Refers to the aORs.

After adjusting for country, employment and household income before the pandemic, those that had been isolated had 1.7 times the odds of reporting reduced access to contraception compared to those who had not isolated (95% CI: 1.3–2.1, *p* < 0.001) (Table 2).

## 4 Discussion

This study highlights how people experiencing income loss and self-isolation may have faced increased barriers to family planning during the pandemic. Personal loss of income and financial insecurity have been linked to reduced access to contraception in other online surveys conducted in the USA (1, 7). Income loss has also been associated with increased odds of not using a preferred contraceptive method (8), which in itself could lead to an increased risk of contraception discontinuation and unintended pregnancy. Loss of income might lead to reduced contraception access due to unaffordability. Direct costs such as the visit or the contraceptive itself could be prohibitive. Indirect costs such as travel and opportunity loss due to missed time at work can also act as barriers. According to a study conducted in Brazil, ethnicity, low education and unemployment also contributed to difficulties with contraceptive methods (9). The link between self-isolation and reduced contraception access has been also described by Kavanaugh and colleagues, who illustrated how those who had tested positive for COVID-19 had 80% higher odds of experiencing healthcare delays (10). These delays, combined with economic insecurity, increase the barriers to contraception, disproportionately affecting already vulnerable groups. To improve access to contraception for these communities, it would be beneficial to have an intersectional approach (11), that considers the overlapping social, economic and cultural factors that contribute to these disparities, including violence, gender-based discrimination, and cultural norms. Violence, including intimate partner violence, could further restrict access to contraception by limiting individuals' ability to seek healthcare or make autonomous reproductive decisions (12). Future research should explore how these factors intersect and compound barriers to contraception access.

This study explores access to contraception during COVID-19 at a multi-country level and includes a relatively large sample size. The study is limited by the data collection method—an online survey—and the sampling method, which was convenience sampling for the majority of the countries involved; only two countries used population-representative sampling. The survey's distribution within sexual health networks likely resulted in respondents being more aware of contraception needs and access, potentially overestimating barriers. Since I-SHARE was an open online survey, the sampling frame is unknown; we cannot determine how many people received the survey link or how many began the survey. This reduces the generalizability of the results, particularly in low-resource settings or among populations with limited internet access (13). Missing data might also represent a source of bias in the study: 18% of the population did not respond to the question on access to contraception, and there were significant differences between those who did and those who did not answer (i.e., they were older, more frequently non-heterosexual and non-cisgender). This may indicate that certain demographic groups were underrepresented, which could affect the robustness of our results. Finally, the association between the exposures of interest may vary significantly according to country; however, it was not possible to calculate measures of association at the country level due to the low number of participants in some countries. Future research should explore these differences in more depth at the country or regional level.

COVID-19 likely exacerbated pre-existing problems and inequalities in contraception access, but it has also highlighted innovative ways to provide contraception and opened new opportunities for more diverse and inclusive services in the future, such as telehealth (14). When planning contraceptive services that may be resilient to epidemics and other health crises, financial support for people who have lost income (15), telehealth and other measures, such as self-administered contraception and postal prescriptions, should therefore be implemented to preserve contraception access and reduce inequalities. More research is needed on the mechanisms that led to reduced access, and on the consequences of reduced contraception access, like increased contraceptive discontinuation: UNFPA has estimated 1.4 million unintended pregnancies in 2020 (16), but there is currently no evidence on the longer-term consequences (17). Finally, future research could explore the role of financial support for those who have lost income during public health crises, and how such support may help mitigate barriers to contraception access.

## Data Availability

The original contributions presented in the study are included in the article/Supplementary Material, further inquiries can be directed to Joe Tucker, jdtucker@med.unc.edu.
