# Supplementary material for: Disparities in adult women's access to contraception during COVID-19: a multi-country cross-sectional survey
Source: Front Glob Womens Health. 2024 Dec 24;5:1235475. doi: 10.3389/fgwh.2024.1235475 (PMC11703851; doi:10.3389/fgwh.2024.1235475)
Supplement: Supplementary file 1 [file Table1.docx]

Supplementary Material

Disparities in adult women’s access to contraception during COVID-19: a multi-country cross-sectional survey

Sara Cavagnis^*^, Rebecca Ryan, Aamirah Mussa, James Hargreaves, Joseph D Tucker, Chelsea Morroni

*** Correspondence:** Sara Cavagnis sara.cavagnis@studio.unibo.it

# Supplementary Figures and Tables

## Supplementary Figures


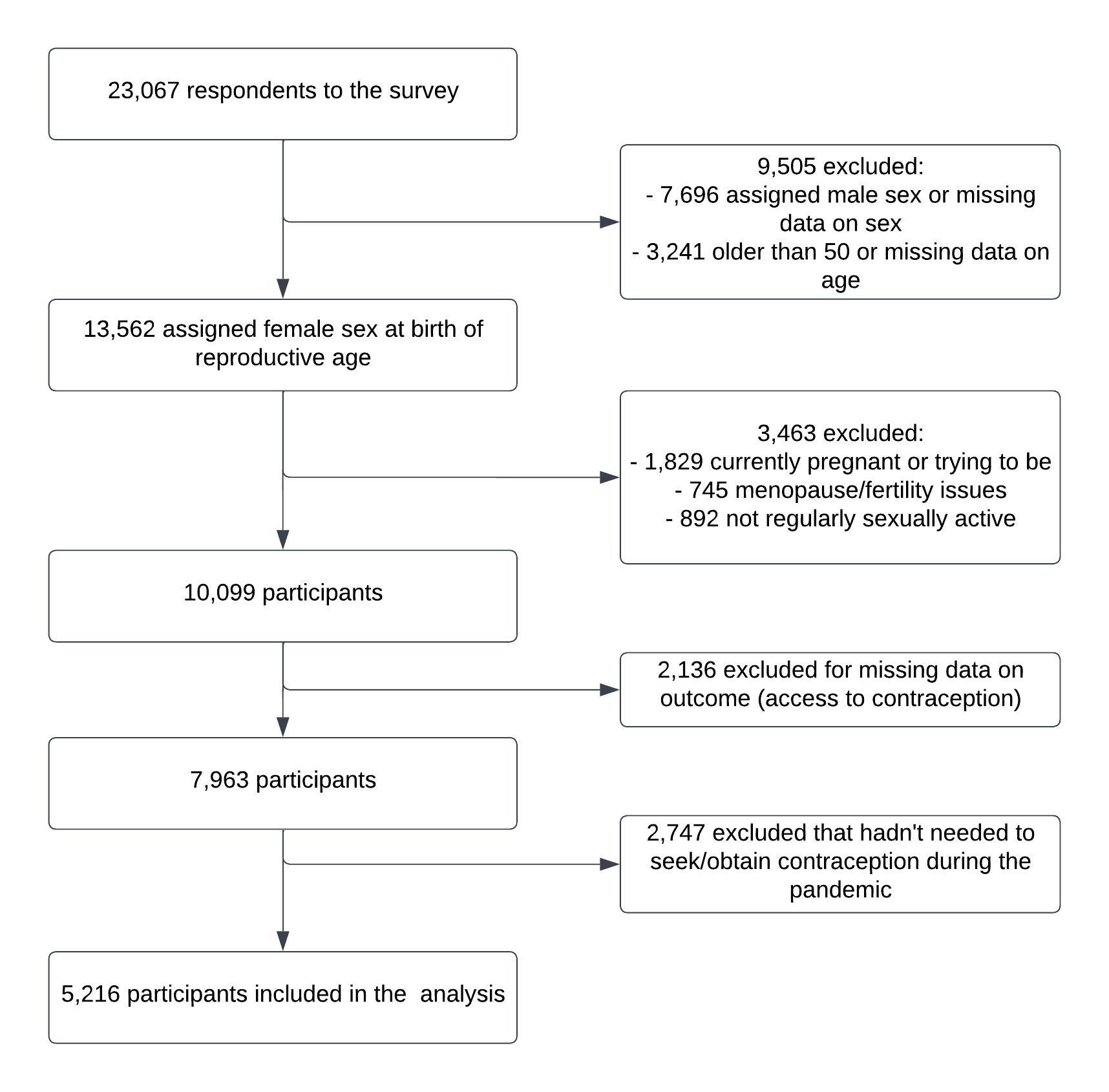


**Supplementary Figure 1.** Flow chart showing how the population included in the final analyses was selected from the original 2020/21 I-SHARE population.

## Supplementary Tables

**Supplementary Table 1.** Number of participants by country, in the original I-SHARE population and in this study. I-SHARE online survey 2020/21.

| **Country** | **Sampling method** | **Number of participants (% of total I-SHARE population)** | **Included in the study (%)** |
| --- | --- | --- | --- |
| Australia | Convenience | 561 (2.4) | 120 (2.3) |
| Canada | Convenience | 163 (0.7) | 31 (0.6) |
| China | Convenience | 827 (3.6) | 152 (2.9) |
| Colombia | Convenience | 2,452 (10.6) | 648 (12.4) |
| Czech Republic | Convenience and population-representative | 662 (2.9) | 262 (5.0) |
| Egypt | Convenience | 31 (0.1) | 4 (0.1) |
| France | Convenience | 1,593 (6.9) | 361 (6.9) |
| Germany | Convenience | 612 (2.6) | 313 (6.0) |
| Italy | Convenience | 329 (1.4) | 119 (2.3) |
| Latvia | Convenience | 1,176 (5.1) | 198 (3.8) |
| Luxembourg | Convenience | 568 (2.5) | 101 (1.9) |
| Malaysia | Convenience | 499 (2.2) | 34 (0.65) |
| Mexico | Convenience | 1,673 (7.2) | 393 (7.5) |
| Moldova | Convenience | 244 (1.1) | 40 (0.8) |
| Mozambique | Convenience | 66 (0.3) | 13 (0.25) |
| Nigeria | Convenience | 231 (1.0) | 4 (0.1) |
| Panama | Convenience | 960 (4.2) | 178 (3.4) |
| Portugal | Convenience | 3,323 (14.4) | 1,029 (19.7) |
| Singapore | Convenience | 566 (2.4) | 29 (0.6) |
| South Africa | Convenience | 29 (0.1) | 5 (0.1) |
| Spain | Convenience | 295 (1.3) | 6 (0.1) |
| Uruguay | Convenience | 696 (3.0) | 236 (4.5) |
| USA | Convenience | 305 (1.3) | 72 (1.4) |
| Argentina | Online panel | 845 (3.7) | 352 (6.75) |
| Botswana | Online panel | 344 (1.5) | 81 (1.55) |
| Kenya* | Online panel | 243 (1.0) |  |
| Lebanon | Online panel | 54 (0.2) | 4 (0.1) |
| Sweden | Online panel | 1,307 (5.7) | 276 (5.3) |
| Uganda | Online panel | 212 (0.9) | 14 (0.3) |
| Denmark | Population-representative | 1,001 (4.3) | 141 (2.7) |
| **Total** |  | **23,067** | **5,216** |

*****Data on age not available: Kenya was excluded from the analyses.

**Supplementary Table 2.** Survey instrument used in I-SHARE (Singapore Version)

| 1. **Selection** | | | | |
| --- | --- | --- | --- | --- |
| 1.1. | Choose your language | List of survey languages |  |  |
| 1.2 | Do you live in Singapore? | 1 Yes  2 No | If 1, go to 1.3 |  |
| 1.2a | Do you live in any of the following countries: | List countries | If in none of the countries, end survey |  |
| 1.3. | How old are you? (in years) | (number) | If <18: end survey.  “Unfortunately, the survey is only for adults aged 18 year of more” If 18 or older, go to informed consent form |  |
| 1.4 | What is your residence status in Singapore? | 1 Singapore citizen  2 Singapore permanent resident  3 Non-Singaporean | If 3, end survey. |  |

| 1. **Socio-demographics** | | | |
| --- | --- | --- | --- |
|  | What is your biological sex? | 1 Woman  2 Man  3 Other (Please specify:______) |  |
| 2.1.a | On a scale of 1 to 5, where 1 is completely as a man and 5 is completely as a woman, with what gender do you identify: | 1 Completely as a man  2 Mostly as a man  3 Equally man and woman  4 Mostly as a woman  5 Completely as a woman  6 Other (please specify: _____) |  |
|  | What best describes your relationship status? (multiple responses possible) | 1 Single, and never had a partner  2 Single, but had a partner previously or currently dating  3 In a relationship but not living together  4 Not legally married but living with a partner  5 Legally married and living together  6 Legally married and not living together  7 Legally married but separated  8 Widowed  9 Divorced  10 Other |  |
|  | How many children do you have, if any? Respond 0 if you don’t have children. | (number) |  |
|  | What is your highest degree of schooling? | 1 No formal education  2 Some primary school  3 Complete primary school  4 Some secondary school  5 GCE ‘O’ Levels  6 GCE “N’ Levels  7 GCE ‘A’ Levels  8 Polytechnic diploma  9 ITE diploma/ NITEC  10 Some university  11 Complete university  12 Postgraduate studies  13 Other |  |
|  | What is your religion? | 1 No religion  2 Buddhism  3 Islam  4 Hinduism  5 Christianity  6 Taoism  7 Sikhism  8 Agnostic  9 Atheist  10 Others (please specify): ______ |  |
|  | What is your race, as reflected on your identity card? | 1 Chinese  2 Malay  3 Indian  4 Others (please specify):___ |  |

Introduction: Since 7 April 2020 the government has issued several circuit breaker measures aimed at social distancing to contain the spread of COVID-19 in Singapore. In this survey we will refer to these measures as the COVID-19 circuit breaker measures.

| 1. **Compliance with COVID-19 social distancing measures** | | | | | | | |  |
| --- | --- | --- | --- | --- | --- | --- | --- | --- |
|  | | How much would you say that you’re following COVID-19 circuit breaker measures? | | 1 not at all  2 a little bit  3 a lot  4 very strictly | |  | |  |
|  | | Were you ever on a leave of absence, stay-home notice, or quarantine order because of symptoms or because you were in close contact with someone with COVID-19 or because you returned from overseas? | | 1 No  2 Yes | |  | |  |
|  | | Were you ever tested for COVID-19? | | 1 No  2 Yes, I tested positive at least once  3 Yes, I have always tested negative | |  | |  |
|  | | How many people lived in your house in the three months before the COVID-19 circuit breaker? A household member is someone who has slept under the SAME roof as you for at least 4 nights per week during the past month | | 1 Number of adults >18 years  2 Number of children 0-9 years  3 Number of teenagers 10-18 years | |  | |  |
|  | | Was your family composition different during the COVID-19 circuit breaker? | | 1 No, the composition of my family was the same  2 The composition of my family was different | | If 1, go to 3.7 | |  |
|  | | How many people lived/ live in your house during the COVID-19 circuit breaker? | | 1 Number of adults >18 years  2 Number of children 0-9 years  3 Number of teenagers 10-18 years | |  | |  |
|  | | What was your employment status the month before the COVID-19 circuit breaker? | | 1 Full time employee (30 hours a week or more)  2 Part time employee (less than 30 hours a week)  3 Self-employed/ business owner  4 Unemployed  5 Informal/ piecemeal work  6 Retired/ pensioned  7 Student  8 Other | |  | |  |
|  | | Since the COVID-19 circuit breaker, has your employment status changed? | | 1 No change: I continue doing the same work and going to the usual job site  2 I keep doing the same work, but from home  3 I keep doing the same work, but partly work from home  4 I am employed and paid but unable to attend or do work  5 I work on reduced time  6 I lost my job/ work/ business  7 I am temporarily unemployed  8 I changed work/jobs | |  | |  |
|  | | Below is an income scale on which 1 indicates the lowest income group and 10 the highest income group in your country. We would like to know in what group your household was in the year before the COVID-19 crisis? Please specify the appropriate number, counting all wages, salaries, pensions and other incomes | | 1 Lowest group  2  3  4  5  6  7  8  9  10 Highest group | |  | |  |
| 3.9a | | What is your housing type? | | 1 1-room HDB Flat  2 2-room HDB Flat  3 3-room HDB Flat  4 4-room HDB Flat  5 5-room HDB Flat  6 Executive HDB Flat/ Maisonette  7 Condominium  8 Terrace, Bungalow or other private landed property  9 Other: _______ | |  | |  |
| 3.9b | | On average, what is your gross personal monthly income (before CPF and tax deductions, if any)? | | 1 not earning an income  2 SGD <1000  3 SGD 1000-SGD 1999  4 SGD 2000 – SGD 2999  5 SGD 3000- SGD 3999  6 SGD 4000- SGD 4999  7 SGD 5000-SGD 5999  8 SGD 6000- SGD6999  9 SGD 7000 – SGD 7999  10 SGD 8000 – SGD 8999  11 SGD 9000- SGD 9999  12 SGD 10000 and above | |  | |  |
| 3.10 | | Since the COVID-19 pandemic, the economic situation of many households has changed. Has this been the case for you? | | 1 Yes the economic situation of my household became worse  2 No, the economic situation of my household stayed the same  3 Yes, the economic situation of my household improved | |  | |  |
| 3.11 | | Have you personally experienced a loss of income? | | 1 Yes, a total loss of income  2 Yes, a partial loss of income  3 No loss of income  4 I had no personal income before COVID-19 | |  | |  |
|  | |  | | **Before the COVID-19 circuit breaker** | | **During the COVID-19 circuit breaker, did this increase or decrease?** | |  |
| 3.12 | | How often did you have a drink containing alcohol? | | 1 Never  2 Monthly or les  3 2-4 times a month  4 2-3 times a week  5 4 or more times a week | | 1 Decreased a lot  2 Decreased a bit  3 Stayed the same  4 Increased a bit  5 Increased a lot | |  |
| 3.13 | | How many standard drinks containing alcohol do you have on a typical day when you are drinking?  A standard drink is typically equivalent to one can of beer OR one glass of wine OR one shot of hard liquor | | 1 1-2  2 3-4  3 5-6  4 7-9  5 10+ | | 1 Decreased a lot  2 Decreased a bit  3 Stayed the same  4 Increased a bit  5 Increased a lot | |  |
| 3.14 | | How often do you have six or more drinks on one occasion? | | 1 Never  2 Monthly or less  3 2-4 times a month  4 2-3 times a week  5 4 or more times a week | | 1 Decreased a lot  2 Decreased a bit  3 Stayed the same  4 Increased a bit  5 Increased a lot | |  |
| 3.15 | | How often do you use cannabis (marijuana, hash, grass)? | | 1 Never  2 Monthly or less  3 2-4 times a month  4 2-3 times a week  5 4 or more times a week | | 1 Decreased a lot  2 Decreased a bit  3 Stayed the same  4 Increased a bit  5 Increased a lot | |  |
| 3.15a | | How often do you use prescription medication to help with sleep or relaxation? (e.g. opiate or benzodiazepam-containing medications like cough syrup, muscle relaxants etc.) | | 1 Never  2 Monthly or less  3 2-4 times a month  4 2-3 times a week  5 4 or more times a week | | 1 Decreased a lot  2 Decreased a bit  3 Stayed the same  4 Increased a bit  5 Increased a lot | |  |
| 3.15b | | If you are currently a smoker, what is the average number of cigarettes you smoke daily? | | Open ended | | Open ended | |  |
| 3.15c | | How often do you use other recreational substances not covered above? | | 1 Never  2 Monthly or less  3 2-4 times a month  4 2-3 times a week  5 4 or more times a week | | 1 Decreased a lot  2 Decreased a bit  3 Stayed the same  4 Increased a bit  5 Increased a lot | |  |
| 1. **Couple and family relationships** | | | | | | | | |
|  | Did you have a steady partner in the three months before the COVID-19 circuit breaker? | | | | 1 No  2 Yes | |  | |
|  | Are you currently still in this relationship? | | | | 1 No  2 Yes | |  | |
|  | Did your relationship end before, during, or after COVID-19 circuit breaker? | | | | 1 Before  2 During  3 After | |  | |
|  | Would you say the end of your relationship was precipitated by COVID-19 circuit breaker? | | | | 1 No  2 Yes  3 Not sure | |  | |
|  | Have you had a new steady partner since COVID-19 circuit breaker? | | | | 1 No  2 Yes | |  | |
|  | What is your sexual orientation? | | | | 1 Asexual  2 Bisexual  3 Gay  4 Heterosexual (straight)  5 Lesbian  6 Pansexual  7 Queer  8 Questioning or unsure  9 Other (specify) | |  | |
|  | During the COVID-19 circuit breaker, is/was your steady partner living with you in the same place? (only those responding 2 to 4.2 or 2 to 4.5) | | | | 1 No, s/he stays elsewhere  2 Yes, the whole time  3 Yes, part of the time | |  | |
|  | In the three months before the COVID-19 circuit breaker, how often did you experience tension in your relationship to your partner/spouse? (Only for those responding 2 to 4.2) | | | | 1 Never  2 Monthly or less  3 2-4 times a month  4 2-3 times a week  5 4 or more times a week | |  | |
|  | How has this changed since the COVID-19 circuit breaker? Only for those responding 2 to 4.2) | | | | 1 Much less tension than before  2 A bit less tension than before  3 About the same amount of tension  4 A bit more tension than before  5 A lot more tension than before | |  | |
|  | In the three months before the COVID-19 circuit breaker measures, how often did you experience tension in your relationship to your children? ***Only for those living with children (2.5)*** | | | | 1 Never  2 Monthly or less  3 2-4 times a month  4 2-3 times a week  5 4 or more times a week | |  | |
|  | How has this changed since the COVID-19 circuit breaker measures? ***Only for those living with children (2.5)*** | | | | 1 Much less tension than before  2 A bit less tension than before  3 About the same amount of tension  4 A bit more tension than before  5 A lot more tension than before | |  | |
|  | In the three months before the COVID-19 circuit breaker, how much would you say your partner provided you with emotional support? Only for those responding 2 to 4.2 | | | | 1 A ot  2 Some support  3 little support  4 No support | |  | |
|  | How has this changed during the COVID-19 circuit breaker? Only for those responding 2 to 4.2 | | | | 1 Much less support than before  2 A bit less support than before  3 About the same amount of support than before  4 A bit more support than before  5 A lot more support than before | |  | |
|  |  | | ***Before*** the COVID-19 social distancing measures | | ***During*** the COVID-19 social distancing measures |  | |  |
|  | Who is doing most of the household work in your household? ***(Only for those living with a cohabiting partner/spouse (i.e. those responding 3 or 4 to 4.3)*** | | 1 I was doing most of the household work  2 My partner did most of the household work  3 My partner and I equally contributed to the household work  4 Most members of the household contributed equally  5 Someone else did most of the household work | | 1 I am doing most of the household work  2 My partner is doing most of the household work  3 My partner and I equally contribute to the household work  4 Most members of the household contribute equally  5 Someone else is doing most of the household work |  | |  |
|  | In your household, who was most in control of household spending? ***Only for those living with a cohabiting partner/ spouse (i.e. those responding 3 or 4 to 4.6)*** | | | | 1 I had most control  2 My partner had most control  3 My partner and I had equal control  4 Someone else than my partner and I had most control | |  |  |
|  | Has your power to control household spending changed because of the COVID-measures relative to your partner/spouse? ***Only for those living with a cohabiting partner/ spouse (i.e. those responding 3 or 4 to 4.6)*** | | | | 1 Yes, I now have more control  2 Yes, I now have less control  3 No, I have the same control | |  |  |

| 1. **Sexual behavior** | | | |
| --- | --- | --- | --- |
|  | Have you ever had a sexual experience?  By ‘sexual experience' we mean any kind of experience that you felt was sexually arousing. It could be kissing, touching, intercourse, masturbation, watching sexually explicit images, or any other form of sex. | 1 No  2 Yes | If 1, go to section 9 |
|  |  | In the three months before the COVID-19 circuit breaker | During the COVID-19 circuit breaker |
|  | How satisfied were you with your sex life.. | 1 Very satisfied  2 Somewhat satisfied  3 Not very satisfied  4 Not at all satisfied | 1 Very satisfied  2 Somewhat satisfied  3 Not very satisfied  4 Not at all satisfied |
|  | How often have you or your partner experienced sexual problems (problems getting an erection, or loss of sexual interest, arousal, orgasm, sexual satisfaction)? (Only those responding 2 to 4.2 or 2 to 4.5) | 1 Never  2 Once  3 Sometimes  4 Often  5 Not applicable | 1 Never  2 Once  3 Sometimes  4 Often  5 Not applicable |

|  | The next questions will ask about sexual behaviours in the three months before and during the COVID-19 circuit breaker. How many times have you… | In the three months before the COVID-19 circuit breaker | ***During*** the COVID-19 circuit breaker measures |
| --- | --- | --- | --- |
|  | Hugged, kissed, held hands with or cuddled with your steady partner? (only for those responding 2 to 4.2 or 2 to 4.5) | 1 Never  2 Monthly or less  3 2-4 times a month  4 2-3 times a week  5 4 or more times a week | 1 Decreased a lot  2 Decreased a bit  3 Stayed the same  4 Increased a bit  5 Increased a lot |
|  | Engaged in sexual activities with your steady partner? By sexual activities we mean oral, vaginal, anal intercourse or touching. (only for those responding 2 to 4.2 or 2 to 4.5) | 1 Never  2 Monthly or less  3 2-4 times a month  4 2-3 times a week  5 4 or more times a week | 1 Decreased a lot  2 Decreased a bit  3 Stayed the same  4 Increased a bit  5 Increased a lot |
| 5.5a | Used a condom when you had sex with your steady partner? (only those who responded 2,3,4,5 to 5.5) | 1 Never  2 Rarely  3 Sometimes  4 Most of the time  5 Always | 1 Decreased a lot  2 Decreased a bit  3 Stayed the same  4 Increased a bit  5 Increased a lot |
|  | Masturbated? | 1 Never  2 Monthly or less  3 2-4 times a month  4 2-3 times a week  5 4 or more times a week | 1 Decreased a lot  2 Decreased a bit  3 Stayed the same  4 Increased a bit  5 Increased a lot |
|  | Had sex with someone who you are not in a long-term relationship with (a casual partner)? | 1 Never  2 Monthly or less  3 2-4 times a month  4 2-3 times a week  5 4 or more times a week | 1 Decreased a lot  2 Decreased a bit  3 Stayed the same  4 Increased a bit  5 Increased a lot |
| 5.7a | Used a condom when you had sex with a casual partner? | 1 Never  2 Rarely  3 Sometimes  4 Most of the time  5 Always | 1 Decreased a lot  2 Decreased a bit  3 Stayed the same  4 Increased a bit  5 Increased a lot |
|  | Sent or received naked/semi-naked pictures or videos? | 1 Never  2 Monthly or less  3 2-4 times a month  4 2-3 times a week  5 4 or more times a week | 1 Decreased a lot  2 Decreased a bit  3 Stayed the same  4 Increased a bit  5 Increased a lot |
|  | Had sex in exchange for money, material goods, favors, drugs, or shelter?  By material goods, we mean things like food, rent, clothes/shoes/cell phones, cosmetics, transport, good marks in school or school fees, or items for your children, your family, or yourself | 1 Never  2 Monthly or less  3 2-4 times a month  4 2-3 times a week  5 4 or more times a week | 1 Decreased a lot  2 Decreased a bit  3 Stayed the same  4 Increased a bit  5 Increased a lot |
|  | Watched sexually explicit videos (pornography)? | 1 Never  2 Monthly or less  3 2-4 times a month  4 2-3 times a week  5 4 or more times a week | 1 Decreased a lot  2 Decreased a bit  3 Stayed the same  4 Increased a bit  5 Increased a lot |
|  | Performed/watched sexual acts before a webcam? | 1 Never  2 Monthly or less  3 2-4 times a month  4 2-3 times a week  5 4 or more times a week | 1 Decreased a lot  2 Decreased a bit  3 Stayed the same  4 Increased a bit  5 Increased a lot |
|  | If some of your sexual behaviors have changed due to COVID-19 social distancing measures, why do you think this happened? | Open answer | |

| Access to condoms | | | | |
| --- | --- | --- | --- | --- |
| 5.17. | Did the COVID-19 circuit breaker measure make it more difficult to access condoms? | 1 No  2 Yes  3 Not applicable - I don’t normally use condoms | If 1 or 3, go to section 6 (women) or 9 (men) |  |
| 5.18. | If yes, what made it difficult to access condoms? | 1 No transport available  2 I am afraid I might acquire COVID-19 and therefore do not want to go to the doctor/health centre/shop  3 Shops are closed  4 Condoms were not in stock in my store  5 I am not able/allowed to leave the house  6 Pharmacy/dispensary closed  7 health centre/clinic has long queues or are not accessible at this time  8 I can no longer afford it  9 I can no longer access free condoms  10 Other… | All men, go to section 9 |  |

| 1. **Access to contraceptives** | | | |
| --- | --- | --- | --- |
| **For women** | | | |
|  | Have you ever been pregnant? | 1 No  2 Yes | If 1, go to 6.3 |
|  | How many times have you been pregnant in your life? | (number) |  |
|  | What best describes your current situation? | 1 Currently pregnant or probably pregnant  2 Currently trying to become pregnant  3 Recently had a baby (during the COVID-19 social distancing measures)  4 Not currently pregnant and don’t wish to be in the near future  5 Cannot have children (fertility issue/ medical issue/ menopause) | If 1, go to section 7  If 2, go to section 9  If 3, go to 7.9  If 4, continue  If 5, go to section 9 |
|  | Have you recently changed your mind about having a child soon because of COVID-19 (only for those who respond 2 or 4 on 6.3) | 1 yes, I have decided to postpone my decision to have a child in the near future  2 Yes, I have decided I want a child sooner  3 No, I have not changed my plans |  |
|  | Are you or your partner currently doing something to avoid or delay a pregnancy, including condoms, contraceptive methods, traditional methods, etc.? | 1 No  2 Yes, all the time  3 Yes, most of the time  4 Yes, sometimes | If 2, 3 or 4 go to 6.7 |
|  | Are you currently pregnant? | 1 No  2 Yes  3 I don’t know | If 2, go to section 7. |
|  | Did you give birth during the COVID-19 circuit breaker measures? | 1 No  2 Yes  3 Partially | If 2, go to 7.9. |
|  | Do you currently use a contraceptive method? | 1 No  2 Yes, all the time  3 Yes, most of the time  4 Yes, sometimes | If 2, 3 or 4 go to 6.7. |
|  | What is the main reason you are not using contraception? | 1 I want to get pregnant in the near future  1 I am not regularly sexually active and don’t need contraceptives  3 I am in menopause / I can’t get pregnant  2 I don't know what is the best method to use  3 I am scared of the side-effects  4 My partner objects  5 Other | Continue to section 8. |
|  | What contraceptive method are you currently using? ***(multiple answers possible)*** | 1 Male/female condom  2 Diaphragm  3 Pills  4 Patch/ring  5 Copper IUD  6 Hormonal IUD  7 Implant  8 Injectables  9 Self or partner sterilization  10 Withdrawal  11 Natural methods (rhythm method)  12 Birth control apps  13 Other… |  |
|  | Have the COVID-19 circuit breaker measures **stopped or hindered** you from seeking or obtaining contraception? | 1 No  2 Yes | If no, go to 6.10 |
|  | What stopped or hindered you from seeking or obtaining contraception? ***(multiple answers possible)*** | 1 No transport available  2 I am too afraid I will get COVID-19 if I would go to the doctor/health centre to get contraceptives  3 I am not able/allowed to leave the house  4 Method not in stock  5 Doctor/health professional not available  6 Pharmacy/dispensary closed  7 I can no longer afford it  8 Health centre/clinic has long queues or is not accessible at this time  9 I did not want anyone to know that I am sexually active in general  10 I did not want anyone to know that I am having sex during the circuit breaker period  9 Other |  |
|  | What services were you using to seek or obtain contraceptive services ***before*** the COVID-19 social distancing measures? ***(multiple responses possible)*** | 1 General practitioner  2 Other private specialist clinics  3 Polyclinic  4 Government hospital  5 Online services  6 Telephone services  7 Over the counter services (pharmacy)  8 Other |  |
|  | What services did you use to seek or obtain contraceptive services ***during*** the period when the COVID-19 circuit breaker measures were in place? (multiple responses possible)? | 1 General practitioner  2 Other private specialist clinics  3 Polyclinic  4 Government hospital  5 Online services  6 Telephone services  7 Over the counter services (pharmacy)  8 Other  9 I did not need to seek or obtain contraceptive services during the COVID-19 social distancing measures |  |
|  | How do you describe your use of contraceptions during the COVID-19 circuit breaker measures? | 1 The same as normal  2 More difficult to use the contraceptives as prescribed (e.g. unable to stick to medication routine or adherence)  3 Easier to use the contraceptives as prescribed (e.g. better able to stick to medication routine or adherence) | Go to section 8 |

| 1. **Access to Reproductive Health services, antenatal care, pregnancy and maternal and child health (only women responding yes to 6.3.)** | | | |
| --- | --- | --- | --- |
|  | How many months have you been pregnant? | 1-9 |  |
| 7.1a | When you found out you were pregnant, what was your reaction? | 1 Very unhappy  2 Somewhat unhappy  3 A little happy  4 Very happy |  |
| 7.1b | Had you planned to become pregnant? | 1 yes  2 Yes, but it was sooner than we planned  3 Yes, but it was later than we planned  4 No |  |
| 7.1c | Did you getting pregnant, in your opinion, have anything to do with the COVID-19 situation? | 1 No  2 Yes, I could not access contraceptives because of COVID-19  3 Yes, I could not access emergency contraceptives because of COVID-19  4 Yes, I needed the money/gifts from a sexual relationship  5 Yes, thre is more idling about in the community because schools and companies are closed  6 Yes, there is more violence and rape in the community  7 Other (specify) |  |
|  | Have you missed or delayed pregnancy health care appointments ***during*** the COVID-19 social distancing measures? (Some providers have been seeing their patients by phone or by video conferencing. We are NOT counting those types of visits as missed.) | 1 No  2 Yes, because I am afraid I may acquire COVID-19 in the hospital/health care centre  3 Yes, because the doctor/nurse cancelled or rescheduled the appointment because of COVID-19  4 Yes, other reason |  |
|  | How satisfied are/were you with your pregnancy health care during the COVID-19 social distancing measures? | 1 not at all satisfied  2 not satisfied  3 neutral  4 a bit satisfied  5 very satisfied |  |
|  | Because of COVID-19, did you feel anxious or depressed during your pregnancy? | 1 No  2 Yes, a bit  3 Yes, a lot |  |
|  | Did you receive information on acquiring COVID-19 during pregnancy? **(multiple answers possible)** | 1 No  2 Yes, from my doctor/midwife  3 Yes, from the media  4 Yes, from other sources |  |
|  | Do you have any concerns regarding your delivery in the following weeks/months? | 1 No  2 Yes, I am afraid I may acquire COVID-19 in the hospital/health care centre  3 Yes, I am afraid I might not know how to get to the hospital  4 Yes, other reason |  |
|  | Where you do plan to deliver your baby? | 1 In the health care centre or hospital  2 At home with a health care worker  3 At home with a traditional birth attendant  4 At home alone  5 Other,… | If 1 or 5, go to section 9 |
|  | Why do you plan to give birth at home? | 1 I am concerned about the risk of COVID-19 in health facilities  2 The facility is closed or cannot provide services  3 I have no access to a facility  4 I prefer to deliver at home | Go to section 9 |
|  | Where did you give birth? | 1 At a hospital or health centre  2 At home with a skilled birth attendant  3 At home alone  4 At home with a traditional birth attendant  5 Other | If 1 or 5, go to 7.11 |
|  | Why did you give birth at home? | 1 I was concerned about the risk of COVID-19 in health facilities  2 The facility is closed or cannot provide services  3 I have no access to a facility  4 I planned to deliver at home |  |
|  | Have you missed or delayed post-natal care appointments as a result of the COVID-measures? (Some providers have been seeing their patients by phone or by video conferencing. We are NOT counting those types of visits as missed.) | 1 No  2 Yes, because I was afraid to go to the health services  3 Yes, because the doctor/nurse cancelled or rescheduled the appointment  4 Yes, other reason |  |

| 1. **Abortion (only women) – POSSIBLY OPTIONAL FOR COUNTRIES WHERE ABORTION IS FOREBIDDEN, THOUGH PREFERRED THAT THIS IS ASKED ANYWAY TO ASSESS UNSAFE ABORTIONS** | | | |
| --- | --- | --- | --- |
|  | During the COVID-19 circuit breaker measures have you been in need of a termination of pregnancy (abortion)? | 1 No  2 Yes | If no, go to section 9. |
|  | Did you have an abortion during the COVID-19 circuit breaker measures? | 1 No  2 Yes, a medical abortion (taking pills, e.g. misoprostol, or herbs)  3 Yes, a surgical abortion  4 Yes, with other methods |  |
|  | Has the COVID-19 situation ***stopped or hindered*** you from seeking or obtaining an abortion? | 1 No  2 Yes | If 1 on 8.2 and 8.3., go to 8.5. |
|  | How did the COVID-19 circuit breaker measures ***stop or hinder*** you from seeking or obtaining an abortion? **(multiple answers possible)** | 1 No transport available  2 I am too afraid I will acquire COVID-19 if I would go to the doctor/health centre to get contraceptives  3 I am not able/allowed to leave the house  4 Method not in stock  5 Doctor/health professional not available  6 Pharmacy/dispensary closed  7 I can no longer afford it  8 Health centre/clinic has long queues or is not accessible at this time  9 Other | Go to section 9. |
|  | What services would you use to obtain an abortion ***before*** the COVID-19 circuit breaker measures? (multiple responses possible)? | 1 I never had an abortion before the COVID-19 social distancing measures  2 General practitioner  3 Other private specialist clinics  4 Polyclinic  5 Government hospital  6 Online services  7 Telephone services  8 Over the counter services (pharmacy)  9 Traditional healer  10 Self-medication  11 Abortion clinic  12 Through a non-governmental organization or civil society organization for abortion  11 Other |  |
|  | What services did you use to obtain an abortion ***during*** the COVID-19 circuit breaker measures? **(multiple responses possible)?** | 1 General practitioner  2 Other private specialist clinics  3 Polyclinic  4 Government hospital  5 Online services  6 Telephone services  7 Over the counter services (pharmacy)  8 Traditional healer  9 Self-medication  10 Abortion clinic  11 Through a non-governmental organization or civil society organization for abortion  12 Other |  |
|  | Did you experience any delays in obtaining abortion care? | 1 No  2 Yes, a few days  3 Yes, 1-2 weeks  4 Yes, 3-4 weeks  5 Yes, more than 4 weeks |  |

| 1. **Sexual and gender-based violence** | | | | | | |
| --- | --- | --- | --- | --- | --- | --- |
| 9.1 | In your everyday life, in the three months before the COVID-19 situation, how vulnerable did you feel for sexual harassment or sexual, physical, or emotional assault by someone who does not live in your house? | | | *1* Not vulnerable at all  2 Little vulnerable  3 Neutral  4 Quite vulnerable  5 Very vulnerable | | |
| 9.2 | In your everyday life, during the COVID-19 situation, how vulnerable did you feel for sexual harassment or sexual, physical or emotional assault by someone who does not live in your house? | | | *1* Not vulnerable at all  2 Little vulnerable  3 Neutral  4 Quite vulnerable  5 Very vulnerable | | |
|  |  | ***In the three months before*** the COVID-19 social distancing measures | | ***During*** the COVID-19 social distancing measures |  | |
| 9.3 | Has a partner tried to restrict (online or phone) contact with your family? | 1 No  2 Yes, once  3 Yes, multiple times  4 Not applicable | | 1 No  2 Yes, once  3 Yes, multiple times  4 Not applicable |  | |
| 9.4 | Has a partner insulted you or made you feel bad about yourself? | 1 No  2 Yes, once  3 Yes, multiple times  4 Not applicable | | 1 No  2 Yes, once  3 Yes, multiple times  4 Not applicable |  | |
| 9.5 | Has a partner not provided money to run the house or look after the children, though they had money for other things? | 1 No  2 Yes, once  3 Yes, multiple times  4 Not applicable | | 1 No  2 Yes, once  3 Yes, multiple times  4 Not applicable |  | |
| 9.6 | Has a partner slapped, pushed, hit, kicked or choked you or thrown something at you that could hurt you? | 1 No  2 Yes, once  3 Yes, multiple times  4 Not applicable | | 1 No  2 Yes, once  3 Yes, multiple times  4 Not applicable |  | |
| 9.7 | Has a partner physically forced you to have perform sexual acts when you did not want to? | 1 No  2 Yes, once  3 Yes, multiple times  4 Not applicable | | 1 No  2 Yes, once  3 Yes, multiple times  4 Not applicable |  | |
| 9.8 | Have you ever performed sexual acts when you did not want to because you were afraid of what your partner might do? | 1 No  2 Yes, once  3 Yes, multiple times  4 Not applicable | | 1 No  2 Yes, once  3 Yes, multiple times  4 Not applicable |  | |
| 9.9 | If yes on any of the **before** COVID-19 questions:  Did you ever talk to someone about the violence experiences you had ***before*** the COVID-19 circuit breaker measures? ***(multiple responses possible)*** | | 1 No  2 Yes, to a relative  3 Yes, to a friend  4 Yes, to a phone or online helpline  5 Yes, to the social services  6 Yes, to the police  7 Yes, to an association  8 Yes, other… | | |  |
|  | Did you ever officially report (i.e. file a complaint) any violence experiences you had ***before*** the COVID-19 circuit breaker measures? | | 1 No  2 Yes | | |  |
|  | If yes on any of the **during/after** COVID-19 questions:  Did you ever talk to someone about the violence experiences you had ***during*** the COVID-19 circuit breaker measures? ***(multiple responses possible)*** | | 1 No  2 Yes, to a relative  3 Yes, to a friend  4 Yes, to a phone or online helpline  5 Yes, to the social services  6 Yes, to the police  7 Yes, to an association  8 Yes, other… | | |  |
|  | Did you ever officially report (i.e. file a complaint) any violence experiences you had ***during*** the COVID-19 circuit breaker measures? | | 1 No  2 Yes | | |  |

| 1. **Optional: Female genital mutilation/cutting and early/forced marriage** | | | |
| --- | --- | --- | --- |
|  | Does early marriage (marriage before the age of 18 years) happen in your community? | 1 No  2 Yes  3 I don’t know | If no, go to 10.6 |
|  | Do you have a child between 10 and 18 years old? | 1 No  2 Yes, one  3 Yes, more than one | If no, go to 10.5 |
|  | Before the COVID-19 circuit breaker measures, did you intend to arrange a marriage for your child(ren) that are between 10 and 18 years old? | 1 No  2 Yes |  |
|  | Did the COVID-19 situation change your plans to arrange a marriage for your adolescent child(ren)? (multiple responses possible) | 1 No  2 Yes, I will arrange the marriage(s) sooner than planned  3 Yes, I will arrange the marriage(s) later than planned  4 Yes, I have cancelled the marriage plans |  |
|  | In general, do you feel that because of COVID-19, girls and boys are at a higher risk of early marriage? | 1 No  2 Yes, somewhat higher risk  3 Yes, much higher risk  4 I don’t know |  |
|  | Is female circumcision practiced in your community? | 1 No  2 Yes  3 I don’t know | If no, go to section 11 |
|  | Do you have a daughter who is at the age that circumcision is generally done? | 1 No  2 Yes | If no, go to 10.10 |
|  | Before the COVID-19 circuit breaker measures, did you intend to circumcise your daughter? | 1 No  2 Yes |  |
|  | Did the COVID-19 situation change your plans to circumcise your daughter? | 1 No  2 Yes, I decide to do the circumcision sooner than planned  3 Yes, I decide to do the circumcision later than planned  4 Yes, I have cancelled the circumcision plans |  |
|  | In general, do you feel that because of COVID-19, girls are at a higher risk of circumcision? | 1 No  2 Yes, somewhat higher risk  3 Yes, much higher risk  4 I don’t know |  |

| 1. **HIV/STI** | | | | |
| --- | --- | --- | --- | --- |
|  | During the COVID-19 circuit breaker measures have you wanted a test for HIV or another sexually transmitted infection? | 1 No  2 Yes | | If 1, skip to 11.5. |
|  | Has the COVID-19 situation ***stopped or hindered*** you from accessing a test for HIV or another sexually transmitted infection? | 1 No  2 Yes | | If 1, go to 11.4. |
|  | How did the COVID-19 circuit breaker measures stop or hinder you from accessing a test for HIV or another sexually transmitted infection? | 1 No transport available  2 Postal services not functioning  3 Pharmacy closed  4 I can no longer afford it  5 Health centre/clinic has long queues or is not accessible at this time  6 Not able/allowed to leave house  7 Health workers not offering/providing HIV/STI testing services anymore  8 Other (Please specify) | |  |
|  | What services would/did you use to obtain a test for HIV or another sexually transmitted infection? (**multiple responses possible**)? | ***Before*** the COVID-19 social distancing measures | ***During*** the COVID-19 social distancing measures |  |
|  |  | 0 Never needed a test before COVID-19  1 GP  2 Polyclinic  3 Government Sexual health specialist clinic (DSC)  4 Private Hospital  5 Public Hospital  6 Anonymous test site  7 Online services  8 Telephone services  9 Over the counter services (pharmacy)  10 Traditional healer  11 Self-medication  12 Other, specify | 1 GP  2 Polyclinic  3 Government Sexual health specialist clinic (DSC)  4 Private Hospital  5 Public Hospital  6 Anonymous test site  7 Online services  8 Telephone services  9 Over the counter services (pharmacy)  10 Traditional healer  11 Self-medication  12 Other, specify |  |
|  | In your life, have you ever tested positive for HIV? | 1 No  2 Yes  3 Prefer not to answer | | If 1 or 3, go to section 12 |
|  | During the COVID-19 circuit breaker measures, were any appointments at your clinic/health centre for HIV treatment or care cancelled? | 1 No  2 Yes | |  |
|  | During the COVID-19 circuit breaker measures, have you missed or delayed an appointment at your clinic/health centre for HIV treatment or care? | 1 No  2 Yes | | If 1, go to 11.9. |
|  | What was the main reason for missing or delaying an appointment at your clinic/health centre for HIV treatment or care? | 1 No transport available  2 I was too afraid I will acquire COVID-19 if I would go to the doctor/health centre for my HIV treatment or care  3 I am not able/allowed to leave the house  4 Doctor/health professional not available  5 Pharmacy/dispensary closed  6 I can no longer afford it  7 Health centre/clinic has long queues or is not accessible at this time  8 Other | |  |
|  | How did the COVID-19 circuit breaker measures affect your adherence to medication for HIV (on a scale from 1 to 5)? | 1 made adherence to ART impossible  2 made adherence more difficult  3 didn't affect my adherence to ART  4 made adherence somewhat easier  5 made adherence to ART much easier  6 I am not taking medication for HIV at this point | |  |
|  | During the COVID-19 circuit breaker measures, have you been worried that you will run out of ART tablets/your HIV medication because of the lockdown? | 1 Very worried  2 A bit worried  3 Not worried | |  |
|  | Have the COVID-19 circuit breaker measures prompted you to disclose your HIV status? | 0 no, I continued to keep my status private  1 no, I had already disclosed my status  2 yes, it forced me to disclose my status  3 yes, although I was planning on disclosing anyway | |  |

| 1. **Optional: Mental health** | | | |
| --- | --- | --- | --- |
|  | **Since the last week:** |  |  |
| 12.2. | I get angry frequently with slight provocation. | Totally agree  Agree  Agree nor disagree  Disagree  Totally disagree |  |
| 12.3. | Does this happen more or less since the start of the COVID-19 circuit breaker? | A lot more  More  About the same  Less  A lot less |  |
| 12.4. | I have felt frustrated with things in general. | Totally agree  Agree  Agree nor disagree  Disagree  Totally disagree |  |
| 12.5. | Does this happen more or less since the start of the COVID-19 circuit breaker? | A lot more  More  About the same  Less  A lot less |  |
| 12.6. | I have felt bored. | Totally agree  Agree  Agree nor disagree  Disagree  Totally disagree |  |
| 12.7. | Does this happen more or less since the start of the COVID-19 circuit breaker? | A lot more  More  About the same  Less  A lot less |  |
| 12.8. | I have worried about my financial situation. | Totally agree  Agree  Agree nor disagree  Disagree  Totally disagree |  |
| 12.9. | Does this happen more or less since the start of the COVID-19 circuit breaker? | A lot more  More  About the same  Less  A lot less |  |
|  | **General**: |  |  |
| 12.10. | I feel frustrated because of the COVID-19 restrictions | Totally agree  Agree  Agree nor disagree  Disagree  Totally disagree |  |
| 12.11. | I am confused about what I can or cannot do due to COVID-19. | Totally agree  Agree  Agree nor disagree  Disagree  Totally disagree |  |
| 12.12. | I am afraid to acquire COVID-19. | Totally agree  Agree  Agree nor disagree  Disagree  Totally disagree |  |
| 12.13. | I experience obsessive or compulsive behaviors with regards to hand washing. | Totally agree  Agree  Agree nor disagree  Disagree  Totally disagree |  |
| 12.14. | I am afraid of touching items outside my house. | A lot more  More  About the same  Less  A lot less |  |
| 12.15. | I cannot stop thinking about the COVID-19 epidemic. | Totally agree  Agree  Agree nor disagree  Disagree  Totally disagree |  |
| 12.16. | I have nightmares about the current situation. | Totally agree  Agree  Agree nor disagree  Disagree  Totally disagree |  |
| 12.17 | I feel that there is enough protective gear (gloves, mouth masks, sterilizing alcohol) available for me. | Totally agree  Agree  Agree nor disagree  Disagree  Totally disagree |  |
| 12.18. | I feel the Government fails to provide enough, adequate and true information concerning the COVID-19 outbreak. | Totally agree  Agree  Agree nor disagree  Disagree  Totally disagree |  |
| 12.19. | If I have to sneeze or cough in my household, I try to hide this from the people around me. | Totally agree  Agree  Agree nor disagree  Disagree  Totally disagree |  |
| 12.20. | If I would be outside and I would have to sneeze or cough, I would try to hide this from the people around me. | Totally agree  Agree  Agree nor disagree  Disagree  Totally disagree |  |
| 12.21. | How would you rate your overall mental health right now? | Poor  Fair  Good  Very good  Excellent |  |
|  | **In the past two weeks, how often have you been bothered by…** |  |  |
| 12.22. | …feeling down, depressed or hopeless? | Totally agree  Agree  Agree nor disagree  Disagree  Totally disagree |  |
| 12.23. | Does this happen more or less since the start of the lockdown? | A lot more  More  About the same  Less  A lot less |  |

| 1. **Optional: Nutrition** | | | |
| --- | --- | --- | --- |
|  | During the COVID-measures, did you worry that your household would not have enough food? | 1 No  2 Yes, but less than before  3 Yes, but not more than before  4 Yes, more than before |  |
|  | During the COVID-measures, were you or any household member not able to eat the kinds of foods you preferred because of a lack of resources? | 1 No  2 Yes, but less than before  3 Yes, but not more than before  4 Yes, more than before |  |
|  | During the COVID-measures, did you or any household member eat less in either the morning or evening meal than you felt you needed because there was not enough food? | 1 No  2 Yes, but less than before  3 Yes, but not more than before  4 Yes, more than before |  |
|  | During the COVID-measures, were your household food stores ever completely empty and there was no way of getting more? | 1 No  2 Yes, but less than before  3 Yes, but not more than before  4 Yes, more than before |  |
|  | During the COVID measures, did you increase your consumption of foods of low nutritional value (e.g. fast food)? | 1 No  2 Yes, a bit  3 Yes, a lot |  |
|  | During the COVID measures, did you increase your food consumption in general? | 1 No  2 Yes, a bit  3 Yes, a lot |  |
